# The alpha subunit of Go modulates cell proliferation and differentiation through interactions with Necdin

**DOI:** 10.1186/s12964-014-0039-9

**Published:** 2014-07-10

**Authors:** Hyunhee Ju, Sujin Lee, Sunghak Kang, Sung-Soo Kim, Sungho Ghil

**Affiliations:** 1 Department of Life Science, Kyonggi University, Suwon 443-760, Republic of Korea; 2 Department of Anatomy, Ajou University, School of Medicine, Suwon 443-721, Republic of Korea

**Keywords:** Cannabinoid, E2F1, G-protein coupled receptor, Yeast two-hybrid

## Abstract

**Background:**

Heterotrimeric GTP-binding proteins (G-proteins) play an important role in mediating signal transduction generated by neurotransmitters or hormones. Go, a member of the Gi/Go subfamily, is the most abundant G-protein found in the brain. Recently, the alpha subunit of Go (Gαo) was characterized as an inducer of neuronal differentiation. However, its underlying molecular mechanisms have remained unclear to date, since the downstream effectors of Gαo are ambiguous.

**Results:**

A neurally differentiated embryonal carcinoma-derived protein (Necdin) was isolated as an interacting partner for Gαo from a mouse brain cDNA library using yeast two-hybrid screening. Interactions between the proteins were confirmed with several affinity binding assays, both *in vitro* and *in vivo*. Necdin interacted directly and preferentially with activated Gαo, compared to wild-type protein. Interestingly, Gαo did not interact with Gαi, despite high sequence homology between the two proteins. We subsequently analyzed whether Gαo modulates the cellular activities of Necdin. Notably, expression of Gαo significantly augmented Necdin-mediated cellular responses, such as proliferation and differentiation. Moreover, activation of type 1 cannabinoid receptor (CB1R), a Gi/oα-coupled receptor, augmented cell growth suppression, which was mediated by Gαo and Necdin in U87MG cells containing CB1R, Gαo, and Necdin as normal components.

**Conclusions:**

These results collectively suggest that Necdin is a candidate downstream effector for Gαo. Our findings provide novel insights into the cellular roles of Gαo and its coupled receptor.

## Background

Heterotrimeric GTP-binding proteins (G-proteins) mediate signaling from G protein-coupled receptors (GPCRs) to intracellular downstream effectors [1]. Binding of agonists to GPCR stimulates G-protein activation by inducing guanine nucleotide exchange from GDP to GTP. This facilitates dissociation of the alpha subunit (Gα) from beta/gamma subunits (Gβγ) of G-protein. Dissociated G-protein subunits, in turn, modulate activation of their downstream effectors.

To date, 21 Gα subunits encoded by 16 genes have been identified [2]. Gαo has been classified as a member of the Gi/o family, owing to its sequence homology with Gαi, and is the most abundant Gα protein in brain tissue. Overexpression of Gαo promotes neuronal differentiation in various cell types, including PC12, N1E115, and Neuro2a [3]–[5]. Previously, we demonstrated that Gαo increases the number of newly forming neurites in an F11 neuroblastoma cell line [6]. Gαo additionally induces activation of Ras-like protein in all tissues (Rit), which triggers Erk-mediated neuronal differentiation in neuro2A cells [7]. These findings collectively indicate that Gαo acts as an inducer of neuronal differentiation in neurogenic cells. However, the downstream effectors for Gαo and related signaling pathways have not been fully elucidated.

Cannabinoids, the major components of *Cannabis sativa Linnaeus* (marijuana), have recently received considerable attention as potential therapeutic agents, owing to their various pharmacological actions, including pain control, tumor regression, neurogenesis, neuroprotection, and anti-inflammatory effects [8]–[12]. Two types of cannabinoid receptors, designated Gi/o-coupled receptors, have been identified: (1) type I cannabinoid receptor (CB1R) cloned in 1990 [13], predominantly expressed in the brain [14], and (2) type II cannabinoid receptor (CB2R) cloned in 1993 [15], mainly expressed in cells of the immune system [16].

Neurally differentiated embryonal carcinoma-derived protein (Necdin) was originally isolated from P19 embryonic carcinoma cells [17]. Necdin, primarily identified as a functional analog of retinoblastoma protein (Rb), acts as a cell growth suppressor [18]. Additionally, Necdin is reported to induce differentiation in various cell types, including neuronal, muscular, and adipose cells [17],[19]–[21]. Necdin interacts with several Rb-interacting proteins, including SV40 large T antigen and adenovirus E1A, and binds directly to the transcription factor, E2F1, to inhibit its function [22]. Similar to Rb, which induces neuronal differentiation by inhibiting E2F1-associated cell cycle progression, ectopic expression of Necdin triggers neuronal differentiation in N1E-115 neuroblastoma cells [23].

In this study, we performed yeast two-hybrid screening to identify downstream effectors for Gαo using a constitutively active form of Gαo as bait from a mouse brain cDNA library. Consequently, Necdin was identified as a Gαo-interacting protein. Interactions between Gαo and Necdin, both *in vitro* and *in vivo*, were further confirmed with several affinity binding assays. Furthermore, activation of Gαo enhanced Necdin activity. Our findings collectively indicate that Necdin is a candidate downstream effector for Gαo.

## Results

### Interactions of Gαo with Necdin *in vitro* and *in vivo*

**Figure 1:**
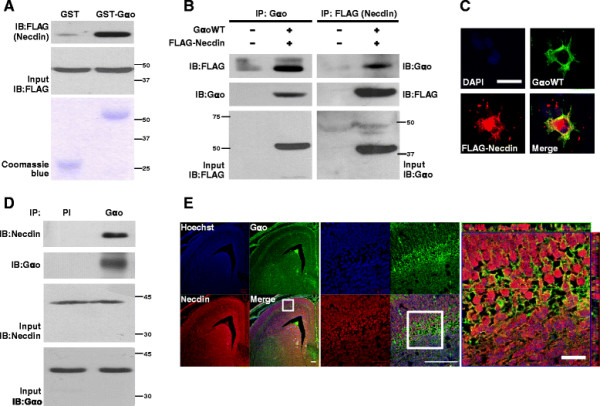
**Gαo interacts with Necdin*in vitro*and*in vivo*. (A)** Beads charged with bacterially expressed GST or GST-Gαo were incubated with 293T cell extracts expressing 10 μg of plasmid encoding FLAG-Necdin. After extensive washing with PBTX buffer, bound proteins were immunoblotted with antibodies against FLAG. Input was loaded with 10% of 293T cell extracts used for the GST pulldown assay, and Coomassie blue staining used to estimate the levels of GST and GST-Gαo proteins. The numbers beside blot indicate size marker (kDa). **(B)** Lysates (500 μg) of 293T cells mock-transfected and expressing GαoWT (3 μg) and FLAG-Necdin (3 μg) plasmids were incubated with antibodies against Gαo or FLAG, as indicated, and the immunoprecipitates immunoblotted with anti-FLAG and Gαo antibodies. Input was loaded with 10% of 293T cell extracts used for the immunoprecipitation assay. **(C)** 293T cells co-transfected with the plasmid encoding GαoWT (0.1 μg) and FLAG-Necdin (0.25 μg) were subjected to immunofluorescence analysis using anti-Gαo and Alexa Fluor 488-conjugated secondary (green fluorescence) antibodies and anti-FLAG and Alexa Fluor 568-conjugated secondary antibodies (red fluorescence). Co-localization of the two types of fluorescence is indicated in yellow in the merged image. The DAPI image represents the cell nucleus. Scale bar = 20 μm **(D)** Newborn rat brain extracts (500 μg) were immunoprecipitated with 1 μg of pre-immune serum (PI) and anti-Gαo antibodies. Immunocomplexes were analyzed by immunoblot analysis using antibodies against Necdin and Gαo. Input was loaded with 10% of brain extracts used for the immunoprecipitation assay. **(E)** Mouse brain (E15.5) section was subjected to immunofluorescence analysis using anti-Gαo and Alexa Fluor 488-conjugated secondary (green fluorescence) antibodies and anti-Necdin and Alexa Fluor 594-conjugated secondary antibodies (red fluorescence). Co-localization of the two types of fluorescence is indicated in yellow in the merged image. The Hoechst image represents the cell nucleus. Scale bar = 100 μm/100 μm/20 μm.

We performed yeast two-hybrid screening to identify Gαo-interacting partners from a mouse brain cDNA library. Necdin protein was isolated using the constitutively active mutant of Gαo as bait, and interactions confirmed with several affinity binding assays. Beads charged with bacterially expressed GST or GST-Gαo proteins were incubated with soluble proteins obtained by detergent extraction of 293T cells transfected with FLAG-tagged Necdin (FLAG-Necdin), and the reaction mixtures probed with antibodies against FLAG. GST-Gαo specifically interacted with FLAG-Necdin (Figure 1A). To determine whether these interactions also occur in the mammalian cellular context, 293T cells were transfected with plasmids encoding wild type of Gαo (GαoWT) and FLAG-Necdin, and the lysates immunoprecipitated and immunoblotted with the indicated antibodies. Consistently, Necdin was identified as an interacting partner of Gαo in mammalian cells (Figure 1B). To investigate whether the two proteins co-localized in 293T cells, cells were transfected with plasmids encoding GoαWT and FLAG-Necdin, and stained with the corresponding antibodies. Confocal microscopy images obtained from the immunofluorescence study indicated co-localization of the two proteins in the cell membrane (Figure 1C). Immunoprecipitation experiments were performed using newborn rat brain extracts to investigate endogenous interactions between the two proteins. Immunocomplexes that precipitated with antibodies against Gαo were analyzed using anti-Necdin antibodies. Necdin proteins were detected in immunoprecipitates of Gαo (Figure 1D). Finally, we performed immunohistochemistry using mouse E15.5 brain to test their expression and co-localization *in vivo*. As shown in Figure 1E, Gαo was mainly expressed in cell membrane and cell process of subplate neuron. Necdin was also expressed in perinuclear region of both cortical plate and subplate neurons. Gαo and Necdin were co-localized in cell membrane of subplate neuron. These results clearly imply that Gαo specifically interacts with Necdin *in vitro* as well as *in vivo*.

### Characteristics of Gαo interactions with Necdin

**Figure 2:**
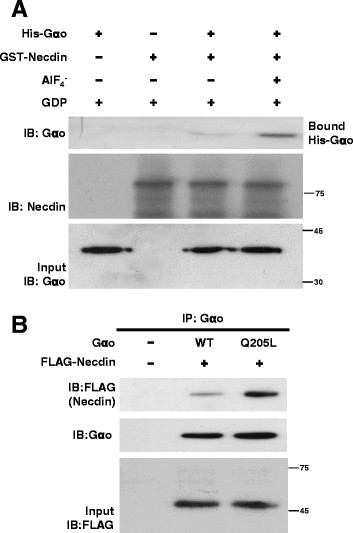
**Necdin interacts directly and preferentially with activated Gαo. (A)** His-Gαo and GST-Necdin fusion proteins were purified from bacterial lysates. His-Gαo (600 nM) was incubated with 30 μM GDP andr AlF_4_^−^ (mixture of 10 mM NaF and 30 μM AlCl_3_), as indicated, for 1 h at 30°C. Purified GST-Necdin (200 nM) was added to the reaction mixture and incubated for an additional 20 min at 20°C. Glutathione-sepharose 4B beads were added to the final reaction mixtures, and the beads analyzed by immunoblot analysis using antibodies against Gαo and Necdin. Input was loaded with 10% of His-Gαo used for the assay. The numbers beside blot indicate size marker (kDa). **(B)** Lysates of 293T cells expressing plasmids either GαoWT (3 μg) or GαoQ205L (3 μg) together with FLAG-Necdin (10 μg) were incubated with anti-Gαo antibodies. Immunocomplexes were analyzed with antibodies against FLAG and Gαo. Input was loaded with 10% 293T cell extracts used for the immunoprecipitation assay.

To determine whether Necdin functions as a downstream effector for Gαo, we incubated with purified His-Gαo and/or glutathione-*S*-transferase (GST)-tagged Necdin (GST-Necdin) proteins in the presence or absence of AlF_4_^−^, an activator of Gα protein. After glutathione-Sepharose beads were added to the reactions, beads were analyzed with antibodies against Gαo and Necdin. Necdin bound directly to Gαo in the presence of AlF_4_^−^ (Figure 2A). Next, we performed a co-immunoprecipitation assay using lysates of 293T cells expressing plasmids encoding the wild-type and constitutively active form of Gαo, together with FLAG-Necdin. Immunocomplexes precipitating with anti-Gαo antibodies were analyzed with immunoblot analysis using antibodies against FLAG and Gαo. Notably, the constitutively active form of Gαo showed higher affinity for Necdin than the wild-type protein (Figure 2B). In view of these results showing that Necdin binds directly and preferentially to the activated form of Gαo, we propose that Necdin is a candidate downstream effector for Gαo.

**Figure 3:**
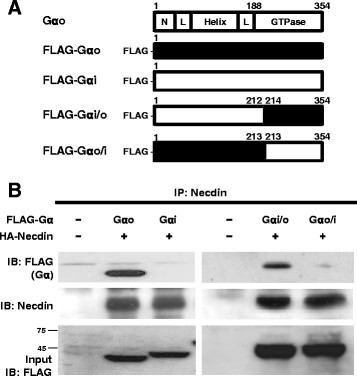
**Gαi does not interact with Necdin. (A)** Schematic presentation of FLAG-tagged Gαo, Gαi, and chimeric proteins, Gαi/o and Gαo/i. A region of the GTPase domain of Gαo was replaced with the corresponding region of Gαi and *vice versa*. **(B)** Lysates of 293T cells expressing plasmids encoding 3 μg of each of the various FLAG-Gα constructs together with 10 μg of HA-Necdin were subjected to the immunoprecipitation assay with anti-Necdin antibodies, and the beads analyzed with antibodies against FLAG and Necdin. Input was loaded with 10% of 293T cell extracts used for the immunoprecipitation assay. The numbers beside blot indicate size marker (kDa).

Since Gαo displays a high degree of sequence homology with Gαi, we examined whether Gαi also interacts with Necdin using lysates of 293T cells co-expressing either FLAG-Gαo or FLAG-Gαi together with hemagglutinin (HA)-tagged-Necdin (HA-Necdin). Cell lysates were incubated with antibodies against Necdin, and the immunocomplexes analyzed with immunoblot analysis using the indicated antibodies (Figure 3B). Interestingly, however, Gαi did not interact with Necdin, unlike Gαo. Next, we employed a chimeric construct [24] of Gαo and Gαi to investigate the Necdin-interacting domain in Gαo (Figure 3A). Lysates of 293T cells co-expressing plasmids encoding FLAG-Gα chimeric proteins and HA-Necdin were subjected to immunoprecipitation analysis with antibodies against Necdin. A chimeric protein, Gαi/o, containing Gαi [1–212] and Gαo [214–354], successfully bound to Necdin, but not Gαo/i containing Gαo [1–213] and Gαi [213–354] (Figure 3B). Our results suggest that Gαo interacts with Necdin via part of the C-terminal region required for GTPase activity.

### Effect of Gαo on the cellular activity of Necdin

**Figure 4:**
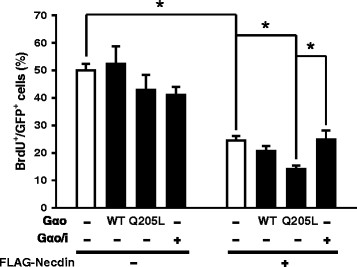
**Gαo enhances cell growth suppression induced by Necdin.** 293T cells were transfected with plasmids encoding various types of Gα (0.5 μg) and FLAG-Necdin (1 μg). To identify transfected cells, we co-transfected with the pEGFP (100 ng). After 24 h of transfection, cells were labeled with 10 μM BrdU for 12 h and stained with antibodies against BrdU and GFP. The extent of BrdU incorporation was assessed in GFP-positive cells. Data are shown as the average ± SE of at least three independent experiments. WT and Q205L indicate wild-type and constitutively active mutant of Gαo, respectively. *, *p* < 0.001.

Necdin is generally expressed in post-mitotic cells, and implicated in various cellular responses, such as cell growth suppression and neuronal differentiation [25]–[27]. To determine whether Gαo augments the growth suppressor activity of Necdin, we performed a BrdU incorporation assay in 293T cells expressing various types of Gα and/or FLAG-Necdin plasmids, indicated. To identify transfected cells, we co-transfected with the green fluorescence protein (GFP) expression plasmid, pEGFP. Cells were observed using fluorescence microscopy, and the proportion of BrdU-positive S-phase cells among GFP-positive cells determined (Additional file 1: Figure S1 and Figure 4). Gα alone (regardless of the types) did not affect the BrdU-positive cell population, compared to control levels. As expected, expression of Necdin promoted a significant decrease in the proportion of BrdU-positive cells. Importantly, cell growth suppression induced by Necdin was augmented by the active form of Gαo, but not Gαo/i.

**Figure 5:**
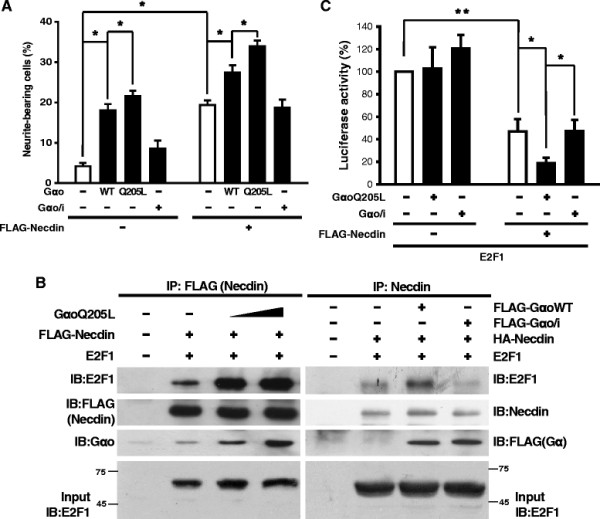
**Gαo promotes Necdin-induced neurite outgrowth. (A)** Neuro2a cells were transfected with plasmids encoding various types of Gα (0.5 μg) and FLAG-Necdin (1 μg). To identify transfected cells, we co-transfected with the pEGFP (100 ng). After 24 h of transfection, cells were serum-starved and observed 30 h later. The proportions of neurite-bearing cells were determined, as described in Materials and Methods section. Data are presented as the average ± SE of at least three independent experiments. *, *p* < 0.001 **(B)** Left panel, Lysates of Neuro2a cells expressing plasmids encoding FLAG-Necdin (10 μg), E2F1 (0.3 μg), and GαoQ205L (1 μg or 3 μg) were subjected to an immunoprecipitation assay using anti-FLAG antibodies, and the beads analyzed with antibodies against E2F1, FLAG, and Gαo. Right panel, Neuro2a cells were transfected with plasmids encoding FLAG-GαWT (10 μg), HA-Necdin (20 μg), and E2F1 (1 μg). Cell lysates were immunoprecipitated with anti-Necdin antibodies. Immunocomplexes were subjected to immunoblot analysis using antibodies against E2F1, Necdin, and FLAG. Input was loaded with 10% of Neuro2a cell extracts used for the immunoprecipitation assay. The numbers beside blot indicate size marker (kDa). **(C)** Neuro2a cells were transfected with the indicated combinations of plasmids encoding GαoQ205L (0.2 μg), FLAG-Gαo/i (0.2 μg), FLAG-Necdin (0.25 μg), E2F1 (0.03 μg), E2F4B-Luc reporter gene (0.1 μg), and β-galactosidase (0.3 μg). The total amount of plasmid DNA used for transfection was maintained by adding pcDNA3. After 48 h, cells were subjected to luciferase and β-galactosidase assays. Luciferase activity was normalized to that of β-galactosidase. Data are presented as the average ± SE of at least three independent experiments. *, *p* < 0.05; **, *p* < 0.001.

Next, we examined whether Gαo affects Necdin-mediated neuronal differentiation. Neuro2a cells expressing the indicated types of Gαo and/or FLAG-Necdin were differentiated via serum starvation. To identify transfected cells, we co-transfected with GFP expression plasmid. At 30 h after serum starvation, cells were observed under a fluorescence microscope, and the percentage of neurite-bearing cells among the GFP-positive population counted (Additional file 2: Figure S2 and Figure 5A). Expression of wild-type and active forms of Gαo promoted neurite outgrowth, whereas Gαo/i did not induce neurite outgrowth. As expected, Necdin promoted neuronal differentiation in the absence of Gαo. Moreover, this effect was synergistically increased by Gαo, but not Gαo/i expression.

Necdin functions by interacting directly with and antagonizing the function of E2F1, a major cell cycle regulatory protein [22],[23]. To determine whether Gαo promotes Necdin::E2F1 binding in Neuro2a, we performed an immunoprecipitation assay using lysates of cells expressing plasmid encoding epitope-tagged Necdin, E2F1 and the indicated types of Gαo (Figure 5B). Immunocomplexes precipitated with antibodies against FLAG or Necdin were analyzed via immunoblot analysis. Interestingly, interactions between Necdin and E2F1 were significantly increased in the presence of Gαo, but not Gαo/i. Next, we investigated whether Gαo enhances the transcription activity of E2F1. Neuro2a cells were transfected with plasmids encoding FLAG-Necdin and different types of Gαo, together with E2F1 and E2F4B-luciferase reporter gene, which contains the E2F1 binding site upstream of the luciferase gene. Luciferase reporter gene activity was increased in the presence of E2F1, and expression of FLAG-Necdin led to a significant decrease in the transcription activity of E2F1, which was synergistically amplified upon co-expression of constitutively activated mutant form of Gαo (GαoQ205L) (Additional file 3: Figure S3 and Figure 5C). However, Gαo/i did not alter the effect of Necdin. Our data clearly indicate that Gαo acts as an enhancer of Necdin activity, supporting the theory that Necdin is a candidate downstream effector for Gαo.

### Effect of CB1R activation on growth suppression in U87MG cells

**Figure 6:**
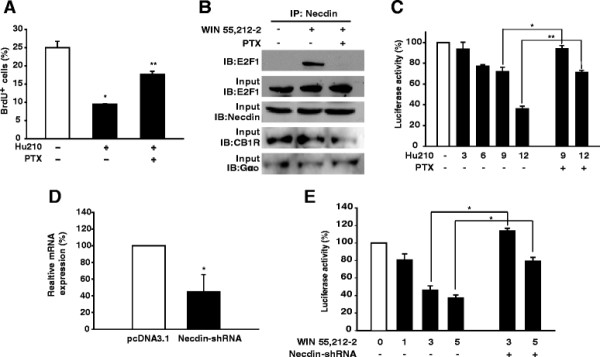
**Cannabinoid inhibits proliferation of U87MG cells. (A)** Serum-starved U87MG cells were treated with 3 μM Hu210 with or without 30 ng/ml of PTX. After labeling with 10 μM BrdU for 12 h, cells were subjected to the BrdU incorporation assay. Data are presented as the average ± SE of at least three independent experiments. *, *p* < 0.001, compared to the control level; **, *p* < 0.001, compared to the level of Hu210 alone. **(B)** Serum-starved cells were treated with 20 μM Win 55,212-2 for 1 h with or without 30 ng/ml PTX, and cell lysates immunoprecipitated with anti-Necdin antibodies. The precipitates were analyzed with antibodies against E2F1. Input was loaded with 5% of U87MG cell extracts used for the immunoprecipitation assay. **(C)** U87MG cells transfected with pGL3-E2F4B-Luc (0.3 μg) and pCMV-β-gal (0.3 μg) were treated with the indicated concentrations (μM) of Hu210 for 6 h with or without 30 ng/ml of PTX. Luciferase activity was normalized to that of β-galactosidase. Data are shown as the average ± SE of at least three independent experiments. *, *p* < 0.05; **, *p* < 0.001. **(D)** U87MG cells transfected with pcDNA3.1 or Necdin-shRNA were subjected to real-time PCR analysis. Data are shown as the average ± SE of at least three independent experiments. *, *p* < 0.05 compare to pcDNA3.1 level. **(E)** The cells transfected with pGL3-E2F4B-Luc (0.3 μg) and pCMV-β-gal (0.3 μg) or Necdin-shRNA (0.2 μg) were treated with indicated concentrations (μM) of Win 55,212-2 for 6 h. luciferase activity was normalized to that of β-galactosidase. Data are shown as the average ± SE of at least three independent experiments. *, *p* < 0.001.

We further investigated the effects of Gαo-Necdin-E2F1 signaling on growth suppression in U87MG cells endogenously expressing Gαo, Necdin, and E2F1. CB1R was additionally expressed as a normal component in cells, and its activation induced the Gi/Go protein-mediated signaling pathway [28]. We examined whether cannabinoid receptor activation increases cell growth suppression and whether this effect is mediated by Gαo-Necdin-E2F1 signaling. We investigated the population of S-phase progression cells using a BrdU incorporation assay. Notably, the number of BrdU-positive cells was decreased in the presence of Hu210. This marked repression was recovered upon pretreatment with PTX, Gi/oα inhibitor (Figure 6A), indicative of Gi/Go protein mediation. Using immunoprecipitation analysis, we additionally examined whether Win 55,212-2, CB1R agonist, affects interactions between Necdin and E2F1, and whether these interactions are blocked by PTX pretreatment. We used Win 55,212-2 instead of Hu210 to activate CB1R. In the presence of Win 55,212-2, we observed Necdin binding to E2F1, which was completely inhibited upon pretreatment with PTX (Figure 6B). The effect of the cannabinoid agonist on Necdin-mediated transcriptional activity of E2F1 was investigated in U87MG cells transfected with the E2F4B-luciferase reporter gene. Treatment with Hu210 induced a dose-dependent reduction in luciferase activity, which was completely abolished upon pretreatment with PTX (Figure 6C). We next tested whether knockdown of Necdin induces recovery of transcriptional activity of E2F1 reduced by CB1R activation. shRNA for Necdin (Necdin-shRNA) expression significantly decrease Necdin expression in U87MG cells (Figure 6D). As shown in Figure 6E, treatment with Win 55,212-2 inhibited transcriptional activity of E2F1 in dose-dependent manner, which was completely abolished by Necdin-shRNA expression. The results indicate that CB1R facilitates U87MG cell growth suppression through the Gαo-Necdin-E2F1 signaling pathway.

## Discussion

In the current investigation, Necdin was identified as a novel binding partner of Gαo. Interactions between Nectin and Gαo were observed both *in vitro* and *in vivo*. Moreover, a region within the C-terminal GTPase domain of Gαo appears to contribute to this interaction. Interestingly, despite high sequence homology of the GTPase domains of Gαi and Gαo, Gαi did not interact with Necdin. Necdin also bound directly to the active form of Gαo with higher affinity than wild-type protein. Accordingly, we propose that Necdin is a candidate downstream effector for Gαo.

To further ascertain whether Necdin acts as a functional effector of Gαo, experiments were performed using two different cell types: (1) 293T and Neuro2a cells transfected with plasmids encoding Gα and Necdin, and (2) U87MG cells endogenously expressing CB1R, which triggers the Gi/o-mediated signaling pathway upon activation. In transfection experiments, the cellular activities of Necdin were significantly enhanced by Gαo, but not Gαo/i. As shown in Figures 4 and 5, Necdin-induced inhibition of cell proliferation and activation of neuronal differentiation were enhanced synergistically by Gαo. Expression of Gαo also led to increased Necdin-E2F1 interactions, representative of Necdin activity. In U87MG cells, activated CB1R inhibited cell proliferation via a PTX-sensitive mechanism, meaning Gi/oα-dependency. Cannabinoid additionally activated Necdin-mediated signaling, including Necdin-E2F1 interactions and E2F1-mediated transcriptional repression. The results collectively suggest that Necdin functions as a downstream effector for Gαo. Nonetheless, the function of Necdin is much less understood in G-protein signaling. We cannot rule out whether Necdin binds to active Gαo thereby prolonging Gβγ signaling. However, the finding that expression of active mutant of Gαo promoted Necdin-mediated cellular activities (Figures 4 and 5) suggests, at least at a first approximation, that Gαo can modulate Necdin functions. Further experiments will be needed to evaluate this using Gβγ inhibitor.

Several lines of evidence support the finding that Gαo modulates neuronal differentiation. Gαo is the most abundant Gα protein in brain tissue and one of the major membrane components of growth cones [4],[29],[30]. Moreover, Gαo is sufficient to enhance neuritogenesis in neurogenic cell lines, including PC12, N1E-115 and Neuro2A [5],[31]. Gαi/o activation induced by CB1R activation inhibits Rap1GAP and activates STAT3 phosphorylation, which, in turn, induces neurite outgrowth in Neuo2A cells [32]. Consistently, our previous studies demonstrated that Gαo functions as an inducer of neuronal differentiation. Gαo overexpression led to new neurite formation via modulation of protein kinase A (PKA) signaling in the F11 neuroblastoma cell line [6]. Further experiments showed that Gαo attenuates nuclear translocation of the catalytic subunit of PKA via direct interactions and augments its cytosolic effects [24]. Additionally, Gαo stimulated the activity of Rit, which induced Erk-mediated neuronal differentiation in Neuro2a cells [7]. Therefore, Gαo mediates signaling of neuronal differentiation by mobilizing various downstream signaling molecules including Necdin.

We postulate that Necdin-mediated neuronal differentiation is attributable to its activity as a strong growth suppressor. Necdin exhibits anti-proliferative activity in various cells and is highly expressed in normal tissues but downregulated in tumor cells [26],[33]. Necdin expression is suppressed by STAT3 whose activation promotes tumor development [34]–[36]. Constitutive expression of STAT3 induces a decrease in Necdin expression at the mRNA and protein levels in tumor cell lines [37]. A previous study demonstrated that a constitutively active form of Gαo induces STAT3-mediated NIH-3 T3 cell transformation [38]. Thus, we considered the possibility that Gαo activation induces cell transformation by enhancing STAT3 activity, in turn, inhibiting Necdin expression. However, in contrast to previous findings, expression of Gαo alone did not affect proliferation in 293T cells (Figure 4). This discrepancy may be explained by the fact that STAT3 activation is an uncommon signaling pathway for Gαo activation in 293T cells, compared to NIH-3 T3 cells.

Considerable evidence supports the theory that cannabinoids are potential therapeutic agents for cancer. Cannabinoids have been shown to inhibit tumor cell proliferation in various cell types, including glioma, pheochromocytoma, prostate, and leukemia cells [39]–[42]. Cannabinoids also inhibit tumor development by blocking angiogenesis and invasiveness [8],[43],[44]. As expected, cannabinoid induced inhibition of cell proliferation in our study. Importantly, cannabinoid activated Necdin-mediated signaling in a PTX-sensitive manner. Although CB1R is coupled with both Gαi and Gαo, Necdin did not interact with Gαi. Therefore, we propose that the CB1R-Necdin signaling pathway is mediated by Gαo, independently of Gαi. The anticancer effects of cannabinoid are mediated via several signaling pathways [45]. We speculate that together with previously reported signaling pathways, the CB1R-Gαo-Necdin-E2F1 pathway contributes to inhibition of cell proliferation in U87MG cells.

Several assay systems are available to measure the cellular activities of Gα proteins. For example, Gαq activity can be measured based on accumulation of inositol phosphate, which is modulated by phospholipase C, a downstream effector of Gαq. The amount of accumulated cAMP represents the activity of adenylyl cyclase, a downstream effector of Gαi and Gαs. However, an effective assay system to measure Gαo activity has not been developed to date owing to the ambiguity of its downstream effectors. Our results showing that Necdin is a candidate downstream effector of Gαo independent of Gαi may facilitate the development of a Necdin-specific assay system for Gαo.

## Conclusions

We have identified a novel downstream effector molecule and signaling pathway for Gαo. Activation of Gαo enhances Necdin-E2F1 interactions, which, in turn, modulate cell differentiation and proliferation. Our current findings present an additional novel signaling pathway to explain the diverse roles of Gαo and cannabinoid receptor.

## Methods

### Construction of plasmids

The pRC/CMV-Necdin plasmid was a generous gift from Dr. K. Yoshikawa (Osaka University, Japan). pcDNA3-E2F1 and pGL3-E2F4B-Luc reporter plasmids were kindly provided by Dr. J. Cheong (Pusan National University, Korea). Plasmids pRC/CMV-GαoWT and pRC/CMV-GαoQ205L encode GαoWT and GαoQ205L of Gαo, respectively [41]. To generate pcFLAG-Necdin, a FLAG-Necdin expression plasmid, pRC/CMV-Necdin, was amplified using the primers 5′-CAT GTC GGA ACA AAG TAA G-3′ and 5′-ATT-TAG-GTG-ACA-CTA-TAG-3′. Amplified PCR products were inserted into pGEM-T Easy plasmid (Promega, Madison, WI, USA). pcFLAG-Necdin was generated by digesting pGEM-T-Necdin with the restriction enzymes, *Not*I and *Xba*I, and ligating the fragment into the corresponding sites of pcFLAG. pcHA-Necdin encoding HA-Necdin was constructed by digesting pcFLAG-Necdin with *Not*I and *Xba*I and ligating the fragment into the corresponding restriction sites of pcHA. pGEX-5X-Necdin, an expression plasmid for GST-Necdin, was generated by digesting pcFLAG-Necdin with *Eco*RI and cloning into the corresponding restriction sites of pGEX-5X (GE Healthcare Life Science, Piscataway, NJ, USA). To generate psi-RNA-hH1ZeoG2-Necdin, Necdin-shRNA plasmid, pcFLAG-Necdin was amplified using the primers 5’-GTA CCT CGC CCG AAG AAC GGA TAG AAG ATC AAG AGT CTT CTA TCC GTT CTT CGG GCT TTT TGG AAA-3’ and 5’-AGC TTT TCC AAA AAG CCC GAA GAA CGG ATA GAA GAC TCT TGA TCT TCT ATG CGT TCT TCG GGC GAG-3’. Amplified PCR products were inserted into psi-RNA-hH1ZeoG2 plasmid (Invivogen, San Diego, CA) using *HindIII* and *ACC65I* restriction enzymes.

### Yeast two-hybrid screening

The bait plasmid (pHybTrp/Zeo-GαoQ205L) was transformed into the yeast reporter cell line, L40, with the mouse brain cDNA library (Clontech, Palo Alto, CA, USA) as recommended by the manufacturer. The methods used for isolation of positive clones are described in a previous report [41].

### Cell culture and transfection

Human embryonic kidney cell line, 293T, mouse neuroblastoma cell line, Neuro2a, and human glioblastoma cell line, U87MG, were maintained in DMEM supplemented with 10% fetal bovine serum (FBS), 100 units/ml penicillin and 100 μg/ml streptomycin. 293T and Neuro2a cells were transiently transfected with the indicated concentrations of plasmids using calcium phosphate and polyethylenimine, respectively. For affinity binding assays, after 48 h of transfection, cells were harvested and extracted with PBTX buffer (PBS containing 5 mM MgCl_2_, 1 mM EDTA, 1% Triton X-100, 5 μg/ml aprotinin, 10 μg/ml leupeptin, 2 μg/ml pepstatin A, and 2 mM phenylmethylsulfonyl fluoride) for 1 h at 4°C with gentle rotation.

### GST pulldown assay

BL21 bacterial cells transformed with pGEX2T-Gαo plasmids [24] encoding GST-Gαo fusion proteins were induced with 0.1 mM IPTG and lysed using a standard protocol. Lysates were incubated with glutathione-Sepharose 4B beads (GE Healthcare Life Science) in PBTX (total volume of 500 μl) for 1 h at 4°C with gentle rotation, and the beads washed extensively with PBTX buffer. 293T cell extracts (500 μg) expressing 10 μg FLAG-Necdin were added to GST-Gαo-bound beads and incubated for 1 h at 37°C (total volume of 500 μl). After extensive washing with PBTX buffer, bound proteins were eluted with SDS sample buffer and subjected to immunoblot analysis with antibodies against FLAG (1:500 dilution, Sigma-Aldrich, St. Louis, MO, USA).

### Direct interactions between His-Gαo and GST-Necdin

Fusion proteins, His-Gαo and GST-Necdin, were purified from BL21 cells using glutathione-Sepharose 4B beads and HisTrap^TM^, respectively [41]. His-Gαo (600 nM) was incubated with 30 μM GDP and AlF_4_^−^ (mixture of 10 mM NaF and 30 μM AlCl_3_), as indicated, in HEMNDL buffer (20 mM Na–HEPES, pH 8.0, 1 mM EDTA, 2 mM MgSO_4_, 150 mM NaCl, 1 mM dithiothreitol, and 0.05% Triton X-100) for 1 h at 30°C. GST-Necdin was added to the reaction at a final concentration of 200 nM, and incubated for an additional 20 min at 20°C. Following the addition of glutathione-Sepharose 4B beads, reactions were incubated for 1 h at 4°C with gentle rotation. Beads charged with proteins were analyzed with the indicated antibodies.

### Immunoprecipitation assay

293T and Neuro2a cells (1.5 × 10^6^ cells/dish) were plated on 100 mm tissue culture dishes and transiently transfected with the appropriate combinations of expression plasmids. U87MG cells (1 × 10^5^ cells/dish) were plated on 100 mm tissue culture dishes. After 24 h, cells were serum-starved with 2% FBS for 20 h. Cells were treated with 20 μM Win 55,212-2, a CB1R agonist, for 1 h in the absence or presence of 30 ng/ml pertussis toxin (PTX, Calbiochem, La Jolla, CA, USA), a Gi/oα inhibitor. Pretreatment with PTX was performed for 20 h prior to Win 55,212-2 application. Newborn rat brain extracts were prepared as reported previously [24]. Cell or brain extracts were pre-cleared by incubating with 20 μl Protein A-Sepharose CL-4B beads (10% slurry) (GE Healthcare Life Science) for 20 min, followed by the addition of 1 μg of the indicated antibodies with gentle rotation for 4 h at 37°C, and subsequently, 50 μl beads. After 2 h of incubation at 4°C, beads were washed with PBTX. Bound proteins were subjected to immunoblot analysis using antibodies against FLAG, Gαo (1:1000, Santa Cruz Biotechnology, Santa Cruz, CA, USA), Necdin (1:500, Cayman Chemicals, Ann Arbor, MI, USA), and E2F1 (1:200, Santa Cruz Biotechnology).

### Immunofluorescence assay

293T cells (1 × 10^3^ cells/coverslip) were plated on coverslips and transfected with expression plasmids for Gαo (0.1 μg) and FLAG-Necdin (0.25 μg). After 48 h, cells were fixed with 4% paraformaldehyde in PBS for 15 min and incubated for 1 h at room temperature with antibodies against Gαo and FLAG. Cells were washed with PBS and incubated with Alexa Fluor 488-conjugated goat anti-rabbit IgG and Alexa Fluor 568-conjugated goat anti-mouse IgG (Life Technologies, Gaithersburg, MD, USA) for 30 min at room temperature. After washing with PBS and counterstaining with DAPI (Vector Laboratories, Burlingame, CA, USA), cells were observed under a LSM510 confocal laser scanning microscope (Carl Zeiss, Thornwood, NY, USA).

### Tissue preparation and immunohistochemistry

Pregnant mice (C57BL/6 J) were killed via cervical dislocation and E15.5 embryos were placed immediately in ice-cold 4% PFA. After overnight fixation, tissues were embedded in paraffin and sectioned to 5-μm thickness. Antigens were retrieved by boiling in 10 mM sodium citrate (pH 6.0) in a microwave oven, the sections were blocked in PBS with 5% normal serum, and probed with primary antibodies against Necdin (1:100; Santa Cruz Biotechnology) and Gαo (1:400). The sections were then incubated with Alexa Fluor 488- or 594-conjugated anti-IgG secondary antibodies and counterstained with Hoechst 33258 (Molecular Probes, Eugene, OR). Fluorescent images were acquired using a Zeiss LSM710 confocal microscope (Carl Zeiss).

### Bromodeoxyuridine (BrdU) incorporation assay

293T and U87MG cells were plated on 6-well plates at a density of 2 × 10^4^ cells per well. 293T cells were transfected with the appropriate combinations of indicated plasmids. To identify transfected cells, we co-transfected with the GFP expression plasmid in all experiments. U87MG cells were serum-starved and pretreated with 30 ng/ml PTX. After 20 h, cells were treated with 3 μM Hu210 (Tocris Bioscience, Ellisville, Mo, USA), a CB1R agonist for 6 h. Both cell lines were labeled with 10 μM BrdU (Sigma-Aldrich), 12 h before harvest. Next, cells were washed with PBS and fixed in 4% paraformaldehyde in PBS for 15 m. After fixation, cells were subjected to immunofluorescence staining against BrdU [46].

### Neurite outgrowth assay

Neuro2a cells were plated on 6-well tissue culture dishes at a density of 3 × 10^4^ cells per well. Cells were transfected with the indicated combinations of plasmids, and 100 ng of pEGFP. After 24 h, cells were serum-starved for 36 h. Fluorescence images were observed with a IX71 fluorescence microscope (Olympus, Tokyo, Japan), and the percentage of neurite-bearing cells among the GFP-positive population calculated. Cell processes greater than cell body in length were counted as neurites.

### Real-time PCR

Total RNA was extracted from cultured cells with the Easy-spin total RNA extraction kit (Intron Biotechnology, Sungnam, Korea), according to the manufacturer’s instructions. First-strand complementary DNA (cDNA) was synthesized using 1 μg of total RNA as the template, 500 ng of oligo (dT), and AccuPower™ RT-Premix (Bioneer, Daejeon, Korea) in a total volume of 20 μl, according to the manufacturer’s recommendation. The relative mRNA expression of Necdin was assessed using TOPreal™ qPCR premix (Enzyomics, Daejeon, Korea) on the CFX96™ PCR System (Bio-Rad, Richmond, CA). Primer sequences used were as follows: Necdin, 5’-GCT CAT GTG GTA CGT GTT GG-3’; and 5’-TGC TTC TGC ACC ATT TCT TG-3’; GAPDH, 5’-TGG GCT ACA CTG AGC ACC AG-3’; and 5’-GGG TGT CGC TGT TGA AGT CA-3’. The Necdin mRNA values were normalized to the amount of GAPDH that was measured.

### Luciferase reporter gene assay

Neuro2a cells were plated on 6-well tissue culture dishes at a density of 3 × 10^4^ cells per well. Cells were transfected with the indicated combination of plasmids, together with 0.1 μg of pGL3-E2F4B-Luc reporter plasmids containing four consensus E2F binding sites [47]. For normalization of transfection efficiency, cells were transfected with 0.3 μg of β-galactosidase expression plasmid (pCMV-β-gal). The total amount of plasmid DNA used for transfection was maintained by adding pcDNA3 (Life Technologies). After 48 h, cell lysates were assayed for luciferase and β-gal activity using the Luciferase Assay System (Promega, Madison, WI, USA), as recommended by the manufacturer. U87MG cells were plated on 6-well plates at a density of 1 × 10^4^ cells per well and transfected with 0.3 μg of pGL3-E2F4B-Luc reporter plasmids using Lipofectamine 2000 (Life Technologies). After 24 h, cells were serum-starved for 16 h and treated with the indicated amounts of Hu210 for 6 h in the absence or presence of 30 ng/ml PTX. Pretreatment with PTX was performed for 1 h before Hu210 application. For knockdown experiment, U87MG cells transfected with pGL3-E2F4B-Luc (0.3 μg) and Necdin-shRNA (0.2 μg) were treated with indicated amount of Win 55,212-2 for 6 h.

## Abbreviations

BrdU: Bromodeoxyuridine

CB1R: Type 1 cannabinoid receptor

CB2R: Type II cannabinoid receptor

FBS: Fetal bovine serum

Gαo: alpha subunit of Go

GαoQ205L: Constitutively activated mutant of Gαo

GαoWT: Wild-type of Gαo

GFP: Green fluorescence protein

GPCR: G protein-coupled receptors

GST: Glutathione-*S*-transferase

Necdin: Neurally differentiated embryonal carcinoma-derived protein

PKA: Protein kinase A

PTX: Pertussis toxin

Rit: Ras-like protein in all tissues

Rb: Retinoblastoma protein

## Competing interests

The authors declare that they have no competing interests.

## Authors’ contributions

HJ, SL, SK and SG designed experiment, HJ, SL and SK performed experiments, and SG and HJ wrote the manuscript. All authors read and approved the final manuscript.

## Additional files

## Supplementary Material

Additional file 1: Figure S1. Gαo enhances cell growth suppression induced by Necdin. 293T cells were transfected with plasmids encoding various types of Gα (0.5 μg) and FLAG-Necdin (1 μg), as indicated. To identify transfected cells, we co-transfected with the pEGFP (100 ng). After 24 h of transfection, cells were labeled with 10 μM BrdU for 12 h and stained with antibodies against BrdU and GFP. Scale bar, 20 μm. Click here for file

Additional file 2: Figure S2. Gαo promotes Necdin-induced neurite outgrowth. Neuro2a cells were transfected with plasmids encoding various types of Gα (0.5 μg) and FLAG-Necdin (1 μg). To identify transfected cells, we co-transfected with the pEGFP (100 ng). After 24 h of transfection, cells were serum-starved and observed 30 h later. Scale bar, 50 μm. Click here for file

Additional file 3: Figure S3. Effect of E2F1 and Necdin on E2F4B-luciferase reporter gene activity. Neuro2a cells were transfected with the indicated combinations of plasmids encoding FLAG-Necdin (0.25 μg), E2F1 (0.03 μg), E2F4B-Luc reporter gene (0.1 μg), and β-galactosidase (0.3 μg). The total amount of plasmid DNA used for transfection was maintained by adding pcDNA3. After 48 h, cells were subjected to luciferase and β-galactosidase assays. Luciferase activity was normalized to that of β-galactosidase. Data are presented as the average ± SE of at least three independent experiments. *, p < 0.001. Click here for file
