# Supplementary figures and images for: The alpha subunit of Go modulates cell proliferation and differentiation through interactions with Necdin
Source: Cell Commun Signal. 2014 Jul 10;12:39. doi: 10.1186/s12964-014-0039-9 (PMC4227020; doi:10.1186/s12964-014-0039-9)

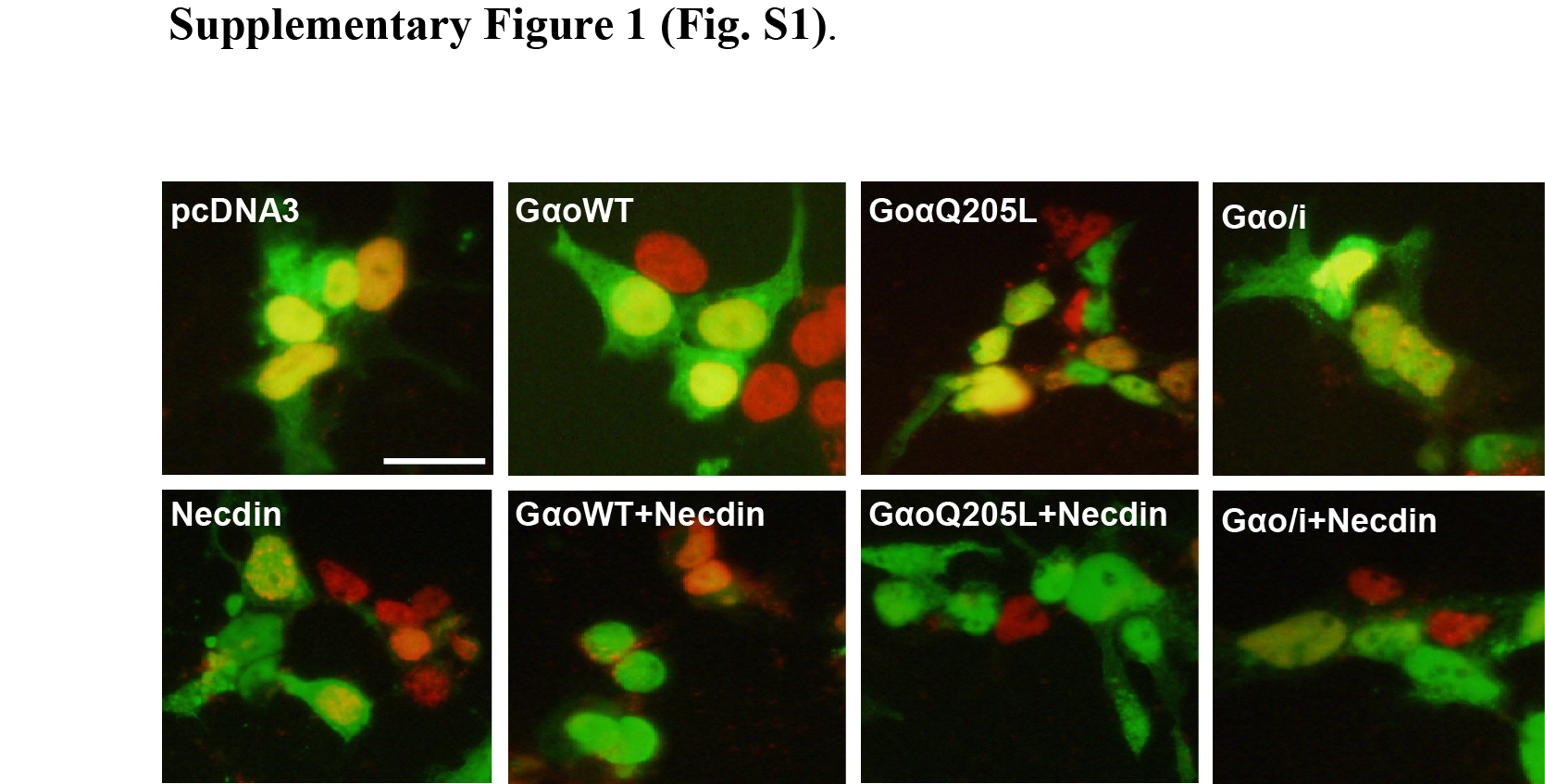

Supplement: Additional file 1: Figure S1. — Gαo enhances cell growth suppression induced by Necdin. 293T cells were transfected with plasmids encoding various types of Gα (0.5 μg) and FLAG-Necdin (1 μg), as indicated. To identify transfected cells, we co-transfected with the pEGFP (100 ng). After 24 h of transfection, cells were labeled with 10 μM BrdU for 12 h and stained with antibodies against BrdU and GFP. Scale bar, 20 μm. [file s12964-014-0039-9-S1.tiff]

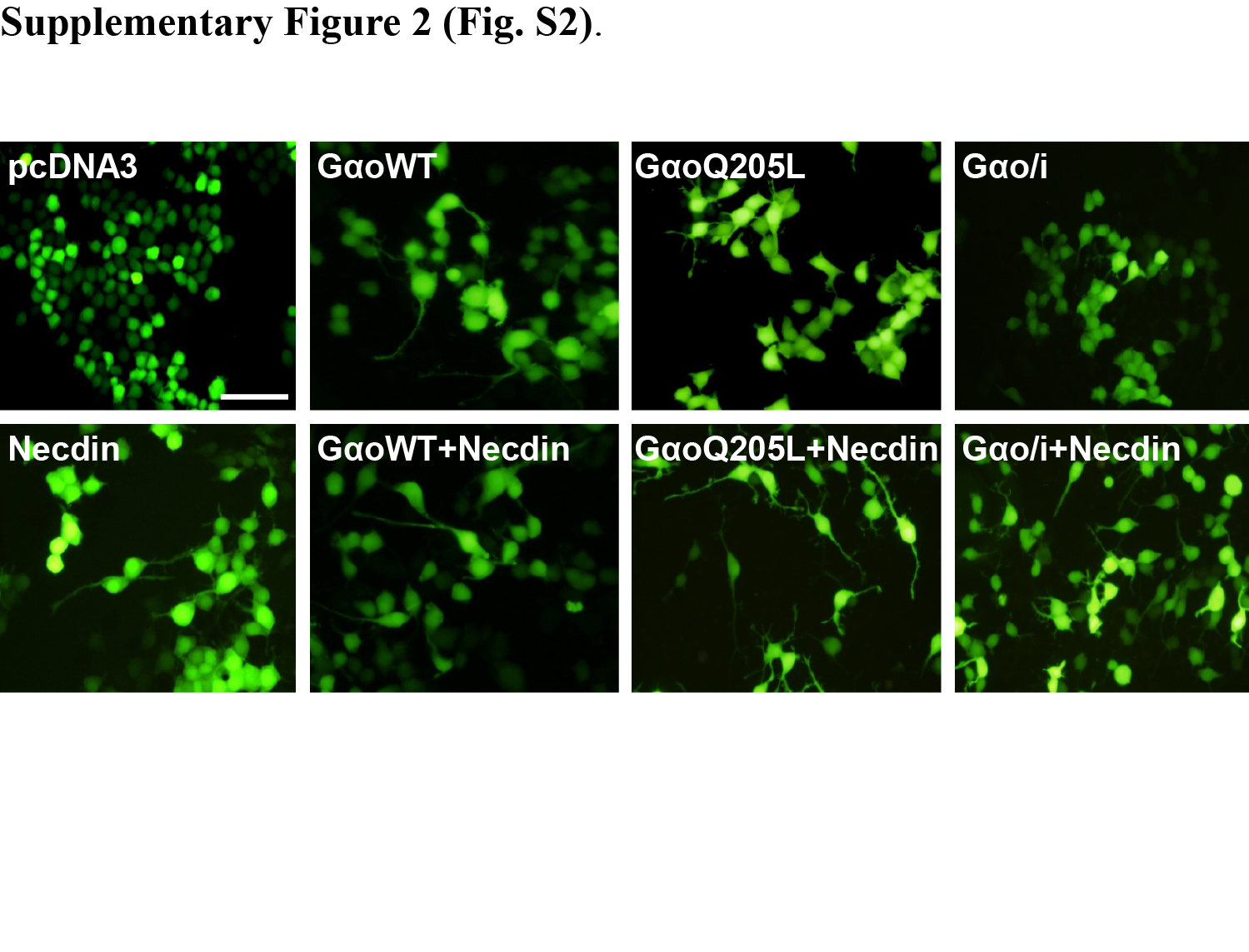

Supplement: Additional file 2: Figure S2. — Gαo promotes Necdin-induced neurite outgrowth. Neuro2a cells were transfected with plasmids encoding various types of Gα (0.5 μg) and FLAG-Necdin (1 μg). To identify transfected cells, we co-transfected with the pEGFP (100 ng). After 24 h of transfection, cells were serum-starved and observed 30 h later. Scale bar, 50 μm. [file s12964-014-0039-9-S2.tiff]

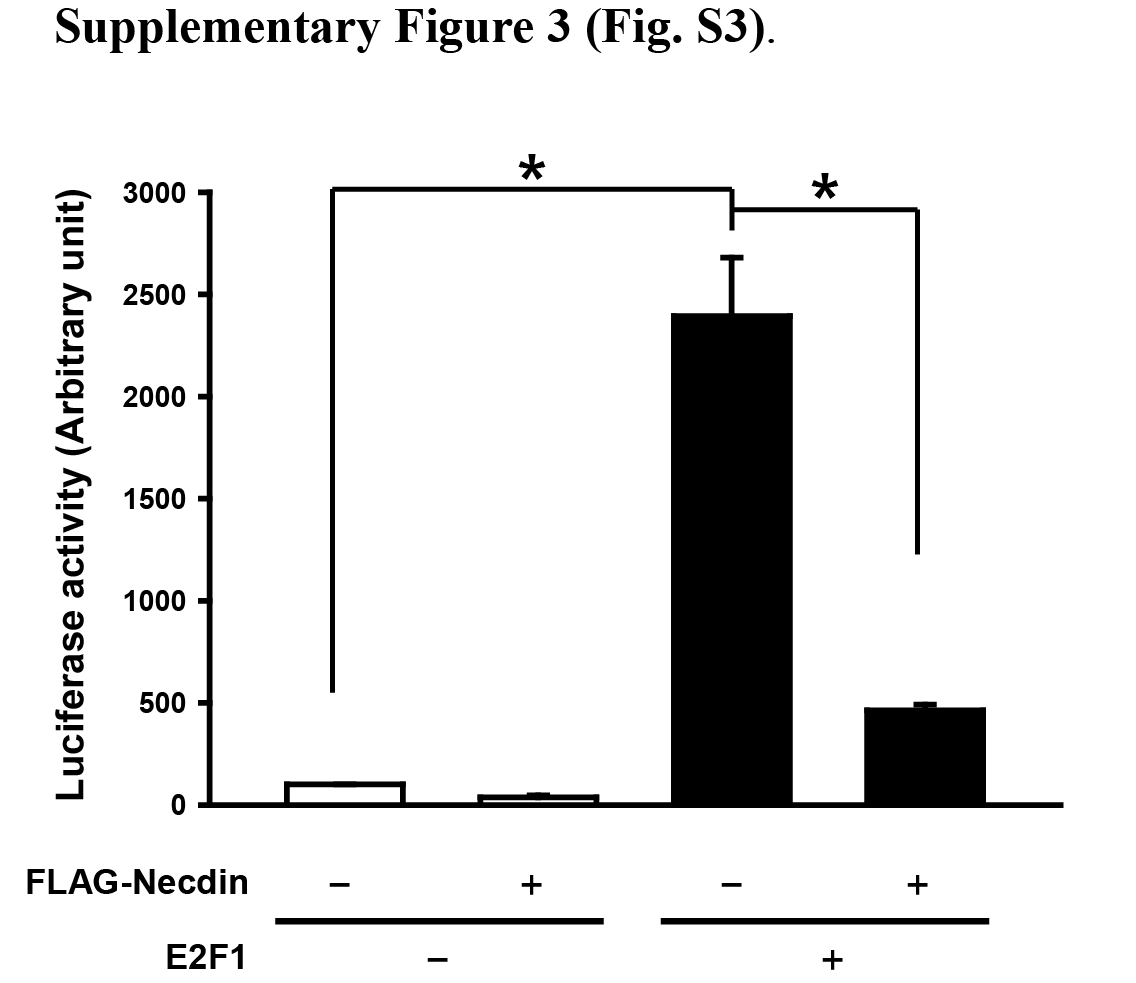

Supplement: Additional file 3: Figure S3. — Effect of E2F1 and Necdin on E2F4B-luciferase reporter gene activity. Neuro2a cells were transfected with the indicated combinations of plasmids encoding FLAG-Necdin (0.25 μg), E2F1 (0.03 μg), E2F4B-Luc reporter gene (0.1 μg), and β-galactosidase (0.3 μg). The total amount of plasmid DNA used for transfection was maintained by adding pcDNA3. After 48 h, cells were subjected to luciferase and β-galactosidase assays. Luciferase activity was normalized to that of β-galactosidase. Data are presented as the average ± SE of at least three independent experiments. *, p < 0.001. [file s12964-014-0039-9-S3.tiff]
